# Medical costs of Swedish nursing home residents at the end of life: a retrospective observational registry study

**DOI:** 10.1186/s12877-024-05043-9

**Published:** 2024-07-04

**Authors:** Dag Salaj, Torbjörn Schultz, Peter Strang

**Affiliations:** 1 Department of Oncology-Pathology, Karolinska Institutet, Stockholm, Region Örebro län, Box 1613, Örebro, S-701 16 Sweden; 2 R&D Department, Sjukhem Foundation, Stockholm, Sweden

**Keywords:** Nursing homes, End-of-life, Palliative care, Health care costs, Costs and cost analysis, Frailty, Hospital frailty risk score, Linear models, Hospitalization, Long-term care

## Abstract

**Background:**

There are many studies of medical costs in late life in general, but nursing home residents’ needs and the costs of external medical services and interventions outside of nursing home services are less well described.

**Methods:**

We examined the direct medical costs of nursing home residents in their last year of life, as well as limited to the period of stay in the nursing home, adjusted for age, sex, Hospital Frailty Risk Score (HFRS), and diagnosis of dementia or advanced cancer. This was an observational retrospective study of registry data from all diseased nursing home residents during the years 2015–2021 using healthcare consumption data from the Stockholm Regional Council, Sweden. T tests, Wilcoxon rank sum tests and chi-square tests were used for comparisons of groups, and generalized linear models (GLMs) were constructed for univariable and multivariable linear regressions of health cost expenditures to calculate risk ratios (RRs) with 95% confidence intervals (95% CIs).

**Results:**

According to the adjusted (multivariable) models for the 38,805 studied nursing home decedents, when studying the actual period of stay in nursing homes, we found significantly greater medical costs associated with male sex (RR 1.29 (1.25–1.33), *p* < 0.0001) and younger age (65–79 years vs. ≥90 years: RR 1.92 (1.85–2.01), *p* < 0.0001). Costs were also greater for those at risk of frailty according to the Hospital Frailty Risk Score (HFRS) (intermediate risk: RR 3.63 (3.52–3.75), *p* < 0.0001; high risk: RR 7.84 (7.53–8.16), *p* < 0.0001); or with advanced cancer (RR 2.41 (2.26–2.57), *p* < 0.0001), while dementia was associated with lower medical costs (RR 0.54 (0.52–0.55), *p* < 0.0001). The figures were similar when calculating the costs for the entire last year of life (regardless of whether they were nursing home residents throughout the year).

**Conclusions:**

Despite any obvious explanatory factors, male and younger residents had higher medical costs at the end of life than women. Having a risk of frailty or a diagnosis of advanced cancer was strongly associated with higher costs, whereas a dementia diagnosis was associated with lower external, medical costs. These findings could lead us to consider reimbursement models that could be differentiated based on the observed differences.

## Background

Despite the differences between health care systems in terms of funding or providing health care to elderly people (common insurance systems, tax financed, out-of-pocket, private insurance), studies from different parts of the world have described high health care costs during the last phase of life [1–3]. Some particular focus has been set to studying the final year of life [3]. Although figures diverge substantially between European and US healthcare systems, many researchers estimate that between 20 and 30% of the whole life’s healthcare resources are spent during the last year of life [2, 4]. The main reasons for this are transitions and hospitalizations in the last six [5, 6] and three [7–9] months of life. Our estimates of the direct healthcare costs in the Stockholm Region (covering approximately 2.4 million inhabitants) are that approximately 10% are spent during the last year of life, which is more in line with the spending for medical care in some comparable European countries [10]. 

End-of-life (EOL) care patterns and health care expenditures differ according to the setting of care. Most of these studies have been performed within the oncological field, where particular attention has been given to different phases (including EOL) as well as to different forms of cancer [11–15]. 

In a study relating cause of death to place of dying and care costs, van der Plas et al. showed that EOL costs differed between different diseases. For persons dying with dementia, nursing home (NH) costs accounted for a greater proportion of healthcare costs in the last year of life than for patients dying with cancer, chronic obstructive pulmonary disease (COPD) or heart failure. In contrast, medical treatment costs dominated for patients with cancer, whereas a mix of hospital treatments and long-term care costs were the main sources of expenditures for patients dying from COPD or heart failure [16]. 

Gender differences in EOL costs have also been described, again within the oncological field. A Norwegian study on direct medical hospital costs [11] reported higher costs for men than for women for nine out of ten non-gender-specific cancer forms, as well as when all cancers were counted together. This approach applied to all studied phases of disease progression. In a separate study [17], using a generalized linear regression model (GLM), the same research team studied cancer patients’ hospital costs in the last year of life and could explain most of the cost differences by the decedents’ age at death and by the proportion of deaths in acute hospital settings.

The studied differences and their causes are of interest and might or might not apply to the care patterns of nursing home (NH) residents, as some characteristics are different. For example, NH residence status has been shown to confound sex differences in Medicare utilization since a much larger proportion of NH residents are women [18]. However, relatively few studies have focused specifically on the EOL expenditure of NH residents [8, 19]; therefore, additional studies are needed.

The Swedish healthcare system is tax-funded for the purpose of ensuring fair and equal distribution to all citizens according to need. There are approximately 14,000 NH residents in the wider Stockholm Region [20]. The staff providing primary care to the resident is employed by the municipality with the exception of the physician, who is provided by the Stockholm Region (formerly known as the Stockholm county council) on a consultative basis. The resident pays for the rent in the NH, the municipality pays for basic costs in the NH (including daily nursing, physiotherapy and occupational therapy, etc.), whereas the Stockholm Region pays for all external healthcare costs (including the NH physician, laboratory and radiology tests, medicines, emergency room visits, in-ward stay if needed, etc.).

Regardless of age, NH residency/care is typically not offered as long as a person can remain in his or her ordinary accommodation when needed with extensive domestic services. The medical conditions of NH residents vary considerably, but cognitive disorders and dementias are found in a majority of residents, while frailty and comorbidities are common [21]. The length of stay in the NH varies considerably. The median NH stay in Sweden was 25 months (21 months without a dementia diagnosis, 30 months with a dementia diagnosis), based on figures during the years 2015–2018 [22]. 

To summarize, there are many last-year-of-life studies on medical costs for people in ordinary accommodations, but less is known about the extent to which NH residents are in need of external medical services and interventions that cannot be offered within NH services. Moreover, neither are the costs known in relation to variables relevant to the NH population, therefore we wanted to investigate whether there are unjustified differences in cost, especially in relation to age and sex, and whether comorbidity and risk of frailty are drivers of external medical costs.

## Methods

We used the guidelines of the Strengthening the Reporting of Observational Studies in Epidemiology (STROBE) to describe our methods and the results [23]. 

### Aims

Our aim was to study direct health care costs (i.e., the costs for the Region) of nursing home residents in their last year of life, adjusted for demographic and clinical factors, including age, sex, comorbidity and risk of frailty. The costs incurred by the municipalities are not included.

### Study design

The registry data used in this retrospective study were retrieved from the Stockholm Region’s central data warehouse (the VAL database) for administrative data. Each clinic and care unit in Region Stockholm must report each patient visit (outpatient as well as inpatient care) with the corresponding World Health Organization International Classification of Diseases (WHO ICD-10) codes to the VAL database and their pay from the Region (formerly: county council) is based on this data. For this reason, the data are close to complete with few missing values. This dataset contains descriptive data such as sex, age, ICD-10 codes, and nationally used KVÅ codes (Klassifikation av Vårdåtgärder, appr. The “National Classification of Care Interventions”, performed in outpatient or inpatient care), and included clinical data such as outpatient visits and periods of inpatient care. The data were collected from nursing home residents (identified via Husläkarregistret, appr “GP register for nursing home residents”, a part of the VAL database) who died in seven consecutive years (2015 to 2021).

### Population

#### The inclusion criterion was as follows:

Nursing home residents aged 65 years or older in the Stockholm Region (covering approximately 2.4 million inhabitants) from 2015 to 2021 were included. During this period, a total of 38,805 residents died.

#### Exclusion criteria

The limited group of persons under the age of 65 years were excluded because they are not representative of the Swedish nursing home population.

### Variables

Reporting to the VAL database is mandatory including all visits to the patients, all the visits to outpatient clinics and emergency wards, all hospitalization periods. The reporting includes mandatory diagnosis setting according to the ICD-10, by the health care professional who is responsible for the visit, i.e. a physician or other registered health professional. The report is mandatory for economic compensation for the respective care unit.

### Outcome variable

Simulated costs (relative costs) are cost estimations that were calculated for the following variables: sex, age groups, comorbidity (Charlson Comorbidity Index [CCI]), risk of frailty (Hospital Frailty Risk Score, HFRS), and main diagnosis (divided into persons with dementia, advanced cancer and others). To control for confounding variables, relative costs were also calculated in multivariable models using generalized linear models (GLMs).

The term SIM cost (Simulated cost) was developed at the Health Care Administration (Hälso- och sjukvårdsförvaltningen, HSF) in Region Stockholm. It is increasingly used to forecast the development of costs in different care areas, for different disease groups, etc., as a basis for budgets and for operational planning.

In brief, the final annual accounts of each care area (somatic specialist care, e.g., hospital inpatient and outpatient care; geriatrics; psychiatry, primary care, other forms of care) were combined with the administrative data in the VAL database. By using different distribution keys, mainly diagnosis-related groups (DRGs), or results from Swedish National Cost Per Patient projects, the cost of each registered care event is estimated via zero-sum calculations. As each care event in the database is connected to an individual, it is feasible to compose, calculate and compare relative costs between major groups and aspects of interest.

In our present study, we applied the cost level in 2019, where the SIM cost was calculated on an annual basis for 60.2 billion Swedish crowns (SEKs). All costs are included in the calculation except costs for drugs that are prescribed on an individual basis (5.7 billion SEK) and costs for central administration (0.8 billion SEK), thus, these costs were not included. As described elsewhere, administrative data where internal average costs are applied with downstream effects are potentially useful in cost studies [24]. 

### Explanatory variables

Our explanatory variables were age, sex, CCI, HFRS, as well as diagnoses of dementia or advanced cancer.

#### CCI

The CCI is based on the International Classification of Disease-10 (ICD-10) codes used to categorize comorbidities (in any medical outcare or incare facility in Stockholm county), and it is often used as a proxy for comorbidity burden [25]. The look-back period used was one year counted backwards from the date when the patient died.

#### HFRS

The HFRS was developed to measure the risk of frailty with the aid of administrative data, and it is based on 109 weighted International Classification of Disease-10 (ICD-10) diagnoses (lookback period: one year). Initially, the HFRS model was created based on a development cohort of more than 22,000 patients with frailty and validated in a cohort of more than 1 million patients, including patients with cancer [26]. In our study, all the ICD-10 codes were retrieved from the VAL database (primary care as well as hospital care). According to the manual, patients with HFRS values of < 5 are judged to have low risk of frailty, whereas 5–15 corresponds with intermediate and > 15 with high risk of frailty.

#### Dementia and advanced cancer

As with the CCI and HFRS, these diagnoses were retrieved from the main or supplemental ICD-10 codes in the VAL database (primary care as well as hospital care). With regard to cancer, only patients with metastatic cancer, or patients with primary malignant brain tumors, or patients with a hematological malignancy at death were included in the comparisons to avoid having cancer in their previous history, as well as persons with indolent tumors.

### Selection bias and dropouts

As reporting to the VAL database is mandatory and directly connected to economic compensation, we estimate that missing data was lower than 1% for most variables.

### Study size

Given that all the nursing home residents who died between 2015 and 2021 were included, no power calculations were performed.

### Analysis and models

As the studied cohort differed in relation to several aspects (women were older but had lower CCI and HFRS scores, etc.), we initially studied the relative costs by applying GLM models for the entire last year of life (regardless of whether the resident had stayed in the nursing home for the entire final year). We thus developed different regression models where our point of departure was a univariable comparison of sex, age and frailty risk score differences, to which we then added a set of variables at the time; thus, we created two multivariable models, thus, we created two multivariable models, which are presented in the Results section.

In Model 3b, adjusted RRs were calculated in a model that included sex, age, and risk of frailty (HFRS). In Model 3c, we added the diagnoses of dementia and cancer to the model as previously described.

Given that not all residents dwelled the whole year in the NH: s, we calculated an alternative cost, which we labeled “stay-day cost”, i.e., cost per day for those days that they actually were nursing home residents. These costs reflect residents’ need for medical care outside the NH (in addition to the municipal care provided within the NH: s).

To compare the relative costs of the GLM models, three separate models were created. We used the same analytical process for the analysis of stay-day costs as for the cost of the last year of life. In Model 5a, univariable data were extracted for each variable. In Model 5b, sex, age and frailty risk groups were entered into an adjusted model, and in the final Model 5c, the diagnoses of dementia or advanced cancer were added to the model.

In the final models 3c and 5c, CCI was not included, as both the CCI and HFRS are based on ICD-10 codes, with a significant correlation.

### Statistical methods and missing data

For the comparison of mean age between men and women, a t test was used. Unadjusted costs were compared using the Wilcoxon rank-sum test, as the data were not normally distributed. Proportions (men and women with cancer and dementia, respectively) were compared using chi-square tests.

GLMs were used in uni- and multivariable models, as these types of statistics are recommended for studies of health care expenditures, where the data are skewed with a small percentage of patients who incur extremely high costs compared to other patients, which implies that distributions of costs are asymmetrical with a long right tail and that heteroscedasticity arises [27]. In the GLM models, we applied a negative binomial distribution and a log-link function [28]. In these models, the exponentiated coefficients are interpreted as rate ratios (RRs), i.e., with an RR of 1.00 indicating no association of a given explanatory variable with increased or decreased values of the response variable, whereas e.g., a RR of 1.10 indicates 10% higher costs than the reference group. There were no missing data for the included individuals; thus, substitutions were not needed. SAS 9.4/Enterprise guide 8.2 was used for statistical analysis.

## Results

The descriptive and clinical data are summarized in Table 1. The total cohort included 38,805 nursing home residents, 63% women and 37% men who died between 2015 and 2021. The mean age at death was 87.5 years, which was greater for women than for men (88.8 vs. 85.2 years, *p* < 0.0001). Women had lower mean Charlson Comorbidity Index (CCI) scores (1.9 vs. 2.7) and lower Hospital Frailty Risk Score (HFRS) values (8.1 vs. 10.2; *p* < 0.0001 in both comparisons). A diagnosis of dementia according to the ICD-10 criteria was registered in 44% of the residents, and 5% of the patients had advanced cancer, defined as cancer with known distant metastases, malignant brain tumor, or a malignant hematologic diagnosis (leukemias, lymphomas or myeloma diagnoses).

**Table 1:** Descriptive and clinical data from 38,805 nursing home residents

Sex	Women	Men	Total
**Sex**, distribution, n (%)	24 394 (63)	14 411 (37)	38 805 (100)
**Age**, mean years (95% CI^1^)^2^	88.8 (88.7–88.9)	85.2 (85.1–85.4)	87.5 (87.4–87.5)
**Age groups**, distribution, n (%)^2^			
65–79 years 80–89 years 90 years or more	2 832 (12) 8 993 (37) 12 569 (52)	3 364 (23) 6 314 (44) 4 733 (33)	6 196 (16) 15 307 (39) 17 302 (45)
**Charlson comorbidity index (CCI)**, n (%)^2^			
0–1 2 or more	12 909 (53) 11 485 (47)	5 486 (38) 8 925 (62)	18 395 (47) 20 410 (53)
**Charlson comorbidity index (CCI)**, linear (95% CI^1^)^2^	1.9 (1.9-2.0)	2.7 (2.6–2.7)	2.2 (2.2–2.2)
**Hospital Frailty Risk Score (HFRS)**, n (%)^2^			
Low risk (< 5) Intermediate risk (5–15)] High risk (> 15)	10 443 (43) 9 710 (40) 4 241 (17)	4 542 (32) 6 159 (43) 3 710 (26)	14 985 (39) 15 869 (41) 7 951 (20)
**Hospital Frailty Risk Score (HFRS)**, linear (95% CI^1^)^2^	8.1 (8.0-8.2)	10.2 (10.1–10.3)	8.9 (8.8–9.0)
**Dementia** (ICD-10 criteria), n (%)^2^	10 655 (44)	6 588 (46)	17 243 (44)
**Advanced cancer**^**3**^ (ICD-10 criteria), n (%)^2^	1 023 (4)	934 (6)	1 957 (5)

^1^95% CI = 95% confidence interval

^2^The difference between men and women was significant, *p* < 0.0001

^3^Advanced cancer was defined as cancer with distant metastases, malignant brain tumor, or a malignant hematologic diagnosis

### Median and mean health care expenditure costs based on last year of life

The mean health care expenditure costs for different subgroups for the last year of life (regardless of the length of stay in nursing homes) are summarized in Table 2. The costs include all medical care outside the nursing home, i.e., mainly primary care, acute hospital care, and care in the geriatric and psychiatric departments. Running costs for nursing home care (mainly provided by assistant nurses and registered nurses) and accommodation costs were not included. As listed in Table 2, the median (mean) cost for men was 106 (192) thousand SEK (based on the 2019 cost level), compared with 56 (130) thousand SEK for women (*p* < 0.0001).

**Table 2:** Median (mean) health care expenditure costs of 38,805 nursing home residents for all medical care provided by the Region (former county council) in their last year of life. The costs were calculated in Swedish crowns (SEK) based on the 2019 cost level

Variable	*N* (%)	Median (IQR) cost per individual, in thousand SEK	Mean (Sd) cost per individual, in thousand SEK	*p* ^1^
**Sex**, distribution				< 0.0001
Women Men	24 394 (63) 14 411 (37)	56 (7-173) 106 (17–257)	130 (204) 192 (288)	
**Age groups**, distribution				< 0.0001
65–79 years 80–89 years 90 years or more	6 196 (16) 15 307 (39) 17 302 (45)	119 (18–319) 84 (11–226) 52 (7-156)	243 (386) 164 (233) 111 (154)	
**Charlson comorbidity index (CCI)**				< 0.0001
0–1 2 or more	18 395 (47) 20 410 (53)	13 (5–80) 152 (62–312)	68 (165) 230 (270)	
**Hospital Frailty Risk Score (HFRS)**				< 0.0001
Low risk (< 5) Intermediate risk (5–15) High risk (> 15)	14 985 (39) 15 869 (41) 7 951 (20)	10 (4–52) 104 (26–218) 231 (120–407)	57 (152) 166 (214) 309 (326)	
**Dementia** (ICD-10 criteria)				< 0.0001
No Yes	21 562 (56) 17 243 (44)	74 (9-238) 72 (10–171)	175 (280) 126 (177)	
**Advanced cancer**^**2**^ (ICD-10 criteria)				< 0.0001
No Yes	36 848 (95) 1 957 (5)	66 (8-187) 303 (148–507)	142 (231) 368 (305)	

*Note* The costs include all medical care outside the nursing home, i.e., mainly primary care, acute hospital care, and care in the geriatric and psychiatric departments. Running costs for nursing home care (mainly provided by assistant nurses and registered nurses) and accommodation costs were not included

^1^The differences between the compared groups were significant

^2^Advanced cancer was defined as cancer with distant metastases, malignant brain tumor, or a malignant hematologic diagnosis

The costs were also affected by age: younger patients had significantly greater median and mean costs (*p* < 0.0001). Moreover, having more comorbidities, as measured by the CCI, or having an intermediate or high risk of frailty, according to the HFRS criteria, were associated with significantly greater costs (*p* < 0.0001 in all comparisons). In contrast, persons with dementia had lower median (mean) costs than did those without dementia (72 (126) vs. 74 (175) thousand SEK; *p* < 0.0001). Those 1957 residents who had advanced cancer had significantly higher median (mean) costs than others did (303 (368) vs. 66 (142) thousand SEK; *p* < 0.0001).

### Multivariable regression models (generalized linear models) for costs

In Model 3a of Table 3, where the mean costs of men during the last year in life were compared univariably with those of women, men had 47% greater costs (RR 1.47 compared to the reference value of 1.00), *p* < 0.0001; however, the association was weakened in the multivariable models with RRs of 1.21 and 1.18.

According to the univariable model, younger age, a lower frailty risk score and a diagnosis of advanced cancer were associated with significantly greater costs, while having a diagnosis of dementia was associated with significantly lower costs. According to the adjusted models (Model 3b and 3c), male sex, younger age, and a higher frailty risk score were significantly associated with greater direct costs.

According to Model 3c, having a dementia diagnosis was also associated with significantly lower costs, and having a cancer diagnosis was associated with significantly greater costs.

**Table 3:** Generalized linear models (GLMs) and last-year health care expenditure costs of nursing home residents. Model 3a: Univariable analysis. Model 3b: Sex, age and frailty risk score (HFRS). Model 3c: Sex, age, frailty risk score (HFRS), dementia status and cancer status

Variable	Model 3a *(Univariable* ^1^ *)* RR (95% CI^3^)	*p* value	Model 3b *(Multivariable* ^2^ *)* RR (95% CI^3^)	*p* value	Model 3c *(Multivariable* ^2^ *)* RR (95% CI^3^)	*p* value
**Men** (ref. women)	1.47 (1.43–1.51)	< 0.0001	1.21 (1.18–1.24)	< 0.0001	1.18 (1.15–1.21)	< 0.0001
**Age** (ref. *≥*90 years)						
65–79 years	2.19 (2.11–2.28)	< 0.0001	2.10 (2.03–2.18)	< 0.0001	1.80 (1.73–1.86)	< 0.0001
80–89 years	1.48 (1.44–1.53)	< 0.0001	1.29 (1.25–1.32)	< 0.0001	1.26 (1.23–1.29)	< 0.0001
**HFRS**^**4**^ (ref. low risk HFRS < 5)						
Intermediate risk (5–15]	2.93 (2.85–3.02)	< 0.0001	2.97 (2.89–3.06)	< 0.0001	3.49 (3.40–3.59)	< 0.0001
High risk (> 15)	5.45 (5.26–5.64)	< 0.0001	5.31 (5.14–5.50)	< 0.0001	7.45 (7.20–7.71)	< 0.0001
**CCI**^**5**^ (ref: CCI 0)						
1	2.25 (2.16–2.33)	< 0.0001			(Not included)^7^	
2 or more	5.85 (5.66–6.05)	< 0.0001			(Not included)^7^	
**Dementia** (ref. no dementia)	0.72 (0.70–0.74)	< 0.0001			0.51 (0.50–0.53)	< 0.0001
**Advanced cancer** ^6^ (ref. no cancer)	2.60 (2.44–2.77)	< 0.0001			2.70 (2.55–2.85)	< 0.0001

^1^Model 3a is a model based on univariable GLM regressions of each variable

^2^Models 3b and 3c are multivariable GLM regression models

^3^CI = Confidence Interval

^4^HFRS = Hospital Frailty Risk Score

^5^CCI = Charlson Comorbidity Index

^6^Advanced cancer was defined as cancer with distant metastases, malignant brain tumor, or a malignant hematologic diagnosis

^7^CCI was not included in the final model, as both the CCI and the HFRS are based on ICD-10 codes, with a significant correlation

### Costs per stay-days

In Table 4, the mean medical costs per day (i.e., the health care costs provided by the region/county council, e.g., for primary care or hospital visits) are summarized in univariable comparisons. The mean costs per stay-day were greater for men, for younger age groups, for those with comorbidities (according to the CCI) or frailty risk (according to the HFRS), and for those with advanced cancer. Patients with an ICD-10 diagnosis of dementia had lower external medical costs than did the other patients according to the univariable comparison.

According to the models, including the final adjusted Model 5c, the mean cost per day was greater for men than for women, for younger NH residents than for older residents, for those with high frailty risk scores (HFRS) than for those with lower scores, and for those with a diagnosis of advanced cancer than for those without a cancer diagnosis. According to all the models (see Table 5), residents with a dementia diagnosis had significantly lower medical costs than residents without a dementia diagnosis did.

Comparing the actual stay-day costs associated with persons dying in the NH (instead of the costs for the whole final year), the differences in costs between men and women were actually even greater.

**Table 4:** Mean nursing home care day costs per stay-day, in Swedish crowns (SEK) of 38,805 nursing home residents for all medical care provided by the Region (former county council). The costs were calculated in SEK based on the 2019 cost level

Variable	*N* (%)	Median (IQR) cost per stay-day, in SEK	Mean (Sd) cost per stay-day, in SEK	*p*
**Age groups**, distribution, n (%)				< 0.0001
65–79 years 80–89 years 90 years or more	6 196 (16) 15 307 (39) 17 302 (45)	222 (40–685) 149 (27–463) 76 (20–303)	801 (2 547) 524 (1 442) 322 (992)	
**Sex**, distribution, n (%)				< 0.0001
Women Men	24 394 (63) 14 411 (37)	87 (20–336) 189 (37–553)	381 (1 250) 642 (1 888)	
**Charlson comorbidity index (CCI)**, n (%)				< 0.0001
0–1 2 or more	18 395 (47) 20 410 (53)	35 (12–162) 272 (78–668)	219 (1 052) 711 (1 818)	
**Hospital Frailty Risk Score (HFRS)**, n (%)				< 0.0001
Low risk (< 5) Intermediate risk (5–15) High risk (> 15)	14 985 (39) 15 869 (41) 7 951 (20)	28 (12–114) 192 (41–464) 426 (168–1027)	157 (781) 516 (1 397) 1 007 (2 411)	
**Dementia** (ICD-10 criteria), n (%)				0.01
No Yes	21 562 (44) 17 243 (56)	112 (23–437) 139 (25–377)	536 (1 720) 406 (1 232)	
**Advanced cancer**^1^ (ICD-10 criteria), n (%)				< 0.0001
No Yes	36 848 (95) 1 957 (5)	113 (23–383) 395 (154–1075)	446 (1 466) 1 073 (2 277)	

*Note* The costs include all medical care outside the nursing home, i.e., mainly primary care, acute hospital care, and care in the geriatric and psychiatric departments. Running costs for nursing home care (mainly provided by assistant nurses and registered nurses) and accommodation costs were not included

^1^Advanced cancer was defined as cancer with distant metastases, malignant brain tumor, or a malignant hematologic diagnosis

**Table 5:** Generalized linear models (GLMs), nursing home care day costs per individual. Model 5a^1^: Univariable analysis. Model 5b: Sex, age and frailty risk score (HFRS). Model 5c: Sex, age, frailty risk score (HFRS), dementia

Variable	Model 5a *(Univariable* ^1^ *)* RR (95% CI^3^)	*p* value	Model 5b *(Multivariable* ^2^ *)* RR (95% CI^3^)	*p* value	Model 5c *(Multivariable* ^2^ *)* RR (95% CI^3^)	*p* value
**Men** (ref. women)	1.69 (1.63–1.74)	< 0.0001	1.33 (1.29–1.37)	< 0.0001	1.29 (1.25–1.33)	< 0.0001
**Age** (ref. *≥*90 years)						
65–79 years	2.49 (2.38–2.60)	< 0.0001	2.17 (2.08–2.26)	< 0.0001	1.92 (1.85–2.01)	< 0.0001
80–89 years	1.63 (1.57–1.68)	< 0.0001	1.38 (1.34–1.42)	< 0.0001	1.35 (1.31–1.39)	< 0.0001
**HFRS**^**4**^ (ref. low risk HFRS < 5)						
Intermediate risk (5–15)	3.29 (3.18–3.40)	< 0.0001	3.18 (3.08–3.28)	< 0.0001	3.63 (3.52–3.75)	< 0.0001
High risk (> 15)	6.41 (6.16–6.67)	< 0.0001	5.88 (5.66–6.12)	< 0.0001	7.84 (7.53–8.16)	< 0.0001
**CCI**^**5**^ (ref: CCI 0)						
1	2.47 (2.37–2.58)	< 0.0001			(Not included)^7^	
2 or more	6.04 (5.81–6.28)	< 0.0001			(Not included)^7^	
**Dementia** (ref. no dementia)	0.76 (0.73–0.78)	< 0.0001			0.54 (0.52–0.55)	< 0.0001
**Advanced cancer** ^6^ (ref. no cancer)	2.41 (2.24–2.58)	< 0.0001			2.41 (2.26–2.57)	< 0.0001

^1^Model 5a is a model based on univariable GLM regressions of each variable

^2^Models 5b and 5c are multivariable GLM regression models

^3^CI = Confidence Interval

^4^HFRS = Hospital Frailty Risk Score

^5^CCI = Charlson Comorbidity Index

^6^Advanced cancer was defined as cancer with distant metastases, malignant brain tumor, or a malignant hematologic diagnosis

^7^CCI was not included in the final model, as both the CCI and HFRS are based on ICD-10 codes, with a significant correlation

## Discussion

We found that the medical care costs, i.e., the Region´s costs for those nursing home residents who needed medical care beyond the care provided by the nursing homes, were not equally distributed. Higher costs were associated with male sex, younger age, comorbidities and/or risk of frailty according to the HFRS criteria. With respect to specific diagnoses, the mean external medical costs were lower for residents with a diagnosis of dementia but significantly greater for those with advanced cancer.

Gender differences in costs have been demonstrated in other contexts. For example, Bugge et al. demonstrated lower last-year-of-life costs for women with cancer, although many of the differences were explained by age, type of cancer and place of death [17]. Studies from other areas, for instance, end-of-life costs of patients with hip fractures, have also shown lower costs for women than men [29]. According to our data, sex remained a significant variable throughout our models, with 18–33% higher costs for men in the multivariable analyses after adjustment for age, frailty risk score and dementia and cancer diagnosis.

A younger age has proven to be a factor significantly associated with higher costs in curative settings, e.g., concerning cancer treatments [30, 31]. This is an expected association, as, e.g., cancer care programs and guidelines have age limits for clinical reasons. Costly treatments that are beneficial in younger age groups could be associated with life-threatening adverse effects in older persons and might therefore not be recommended. Ideally, such age-related differences are not expected to occur in nursing home settings, as the actual medical expenditure should be based on a resident´s medical condition and care needs, not on age. The indications for referrals to Swedish nursing homes are the same regardless of age: persons are offered accommodations in nursing homes only if they have extensive needs for help with daily activities (ADLs) combined with basic needs for medical care or if they suffer from severe dementia. Therefore, those external medical needs that cannot be met by the nursing home staff should be similar, regardless of age. For this reason, the age-related differences in care costs were surprising. However, some of the differences related to gender and age might be mediated by marital status, as being male and of younger age increase the chance of a living partner. Living with a partner possibly increases the threshold for nursing home admissions, and a delayed admission is probably associated with poorer health and, consequently, higher care costs. Unfortunately, marital status is not included in the VAL data bases.

Comorbidities and/or frailty are commonly used as covariable parameters in clinical and cost studies; however, they are not frequently used as primary independent variables, although some studies have been conducted in different settings, with costs or health care utilization as outcomes [32–34]. Of particular interest to our NH setting is an analysis by Mori et al. in which direct health care costs and long-term care costs were analyzed on the basis of the Charlson comorbidity index (CCI), confirming an association between multimorbidity and health care costs as well as with higher long-term care requirements [35]. In our study, we included the CCI in the univariable comparisons but not as a variable in the multivariable analyses, as the CCI and HFRS are both dependent on documented and partly similar or identical ICD-10 diagnoses. For the purpose of this study, we preferred to include HFRS, as frailty is increasingly discussed within geriatrics and gerontology [21].

According to our data, having comorbidities according to the CCI (univariable data) and especially having a risk of frailty according to the HFRS (uni- and multivariable data) were both associated with significantly greater care costs. According to the frailty risk score, 20% of the NH decedents who were identified as belonging to the high-risk group had costs that were more than seven times greater than those belonging to the low-risk group.

In a previous study [21], we demonstrated a significant association between the risk of frailty and a high rate of referral and hospitalization. The need for hospital care constitutes a large proportion of the costs in late life. Obviously, these patients had care needs that were not met with the current staffing: in a typical Swedish nursing home, more than 90% of the staff consists of assistant nurses or persons with even less formal care competence, and physicians are not hired by the communities but are employed by the Regions and work on a consultative basis. It is possible that changes in resource allocation through higher staffing with registered nurses and greater access to physicians could reduce the acute use of hospital care.

Moreover, today, the municipalities and the Region have different medical records for the same patient. Therefore, other measures could include more effective teamwork through collaborative medical records, more active joint care planning and documentation, and sharing consultation information. A greater focus on preventive care in the NH setting has the potential to reduce avoidable, costly hospitalizations [36]. 

Dementia and other forms of cognitive failure are commonplace in nursing home residents. According to our data, having a diagnosis of dementia was associated with lower medical care costs for the Region. Whether this reflects a true, lower need in this patient group cannot be assessed from our registry data. Swedish patients with dementia typically live 7–8 years after dementia diagnosis. Admission to a NH is usually preceded by a high degree of social care intervention and a high burden on relatives but not necessarily by a high external medical health care need, e.g., in acute hospitals. The care trajectory of patients with late-stage dementias can thus, from a medical perspective, often be rather calm and associated with clear and well-accepted care directives. It is also important to distinguish these lower medical costs from the full care costs of the NH staff, which are attributed to the municipality.

However, the greatest difference in terms of specific diagnoses was observed for persons with advanced cancer whose medical care costs were more than double the cost for other residents. Again, we cannot explain this great difference based on our registry data, but the difference mentioned above merits further investigation to assess whether this limited group of nursing home residents (5%) would benefit from special nursing homes with an oncologic profile or at least from greater competence in the palliative care of elderly cancer patients in this special setting.

These data could merit further studies of the differences in costs related to frailty among NH patients. From the perspective of health care provision, these cost differences could lead to the consideration of reimbursement models that could be differentiated based on the degree of comorbidity, frailty risk score or specific and known costly diagnoses. Municipal/NH providers could also consider NH settings specialized in cancer patients and/or patients with care needs that do not motivate specialized palliative hospital care but still demand greater resource usage. To the best of our knowledge, such NHs do not exist in Sweden today. In contrast, NHs specialized in dementia and (to some extent) other psychogeriatric disorders are rather common.

### Strengths and limitations

This study covered all direct medical care costs of NH decedents over a longer study period. The size and complete data coverage contributed to the reliability of the relative comparisons studied. However, all costs incurred by the municipalities are excluded. This limitation is because of the way that NH care is organized in Sweden, with the indirect costs of the care belonging to the municipalities. Future studies, covering the full costs (thus both from the municipalities and regions) would add value to the analysis. Initiatives are currently being taken to create data warehouses that would include all these informations.

Even though reporting of data to the VAL is close to 100% as the economic compensation to respective care unit is based on each reported individual, we cannot rule out some degree of selection bias, including registration errors and automatized registrations, or changes in preferred and registered ICD-10 diagnoses over time.

The study is also limited by the use of registry data. The reason for the registries using simulated costs is that the Swedish healthcare is financed through taxes, there is thus no need for detailing every single cost to an end user (insurance company or patient). We cannot from this study determine whether the cost differences were medically motivated or if they showed inequalities in the given care.

In addition, our study does not include marital status or cohabitation status. Marital status has previously been shown to have significant effects and even been suggested to be a key mediator of sex differences in EOL care [37].

A limitation using retrospective studies is that we already possess the end result, so we know that the patient died. This is a privilege not bestowed upon the clinician, who has to make clinical decisions based on many uncertainties. We cannot fully judge from the analysis the reasons behind the decisions to take clinical actions, to perform or abstain from investigations or referrals.

Studies on decedents have in some cases been criticized [38] as there are possible sources of bias in retrospective registry studies. E.g., the retrospective design may influence subject selection and often, “cases” are compared with controls who are not “cases”. In our study, these problems were minimized, as we did not perform a case-control study, but all NH residents were included and we performed intra-group analyses, rather than comparison with “non-cases”. Variables such as age might influence costs as elderly patients, in contrast to younger patients, may be excluded from expensive treatments e.g., in cancer settings. However, nursing home residents are not candidates for such treatments, regardless of age.

## Conclusions

We found differences in costs with regard to sex, age, and frailty risk scores that cannot be fully explained and seem unmotivated, as admission to NHs are based on care needs, not on variables such as age or sex. We also found differences with regard to suffering from dementia and/or advanced cancer. These costs might be partially explained by the clinical and healthcare trajectories of the patients with these diagnoses. Our data can be used as a basis for future healthcare planning and should be generalizable to countries with similar services, where NHs are tax-funded and based on the need for care, not age.

## Data Availability

The dataset contains information that, although pseudonymized through the encryption of personal identity numbers, could be used to identify individuals through the combination of parts of the dataset, such as the date of death. The dataset is therefore subject to ethical and legal restrictions on public sharing. We cannot share data at the individual level because such data are not permitted according to the laws applied in Sweden. However, the aggregated data generated, used and analyzed during the current study are available from the corresponding author upon reasonable request.

## Abbreviations

COPD: Chronic obstructive pulmonary disease
CCI: Charlson comorbidity index
EOL: End of life
GLM: Generalized linear model
HFRS: Hospital frailty risk score
NH: Nursing home
